# Editorial: The Transition Era to New Sequencing Technologies and Their Application to Integrative Omics in Molecular Surveillance

**DOI:** 10.3389/fgene.2022.840782

**Published:** 2022-02-16

**Authors:** Martin Hölzer, Alejandra Escobar-Zepeda, Jörg Linde, Fabian Horn

**Affiliations:** ^1^ Methodology and Research Infrastructure, MF1 Bioinformatics, Robert Koch Institute, Berlin, Germany; ^2^ European Bioinformatics Institute (EMBL-EBI), Cambridge, United Kingdom; ^3^ Institute of Bacterial Infections and Zoonoses, Friedrich-Loeffler-Institut, Jena, Germany; ^4^ Federal Office of Consumer Protection and Food Safety (BVL), Braunschweig, Germany

**Keywords:** molecular surveillance, sequencing, microbial genomics, genomics, viruses

The current COVID-19 pandemic highlights the importance of novel molecular technologies and methods for rapid pathogen control (Hufsky et al., 2021). In just a few days, South African researchers recently succeeded in sharing crucial sequence information about another worrisome SARS-CoV-2 variant, now called Omicron - helping several countries to monitor and contain this viral variant more quickly (Abdool Karim and Abdool Karim, 2021; Torjesen, 2021). This showcase illustrates that the broad application of sequencing technologies has significantly expanded our portfolio to analyze microbes and the responses of their hosts to health and disease (Gardy and Nicholas, 2018; Gwinn et al., 2019). Researchers analyze isolates, metagenomic and metatranscriptomic samples from various environmental settings, including human, animal, and agricultural environments. The possibility to perform real-time sequencing and data analysis opens up entirely new applications in the field of molecular surveillance and outbreak analysis (Wang et al., 2021). We never before had access to such quantity and quality of tools for molecular monitoring of (emerging) pathogens, mobile genetic elements, virulence and resistance factors, and antibiotic resistance genes.

**FIGURE 1:**
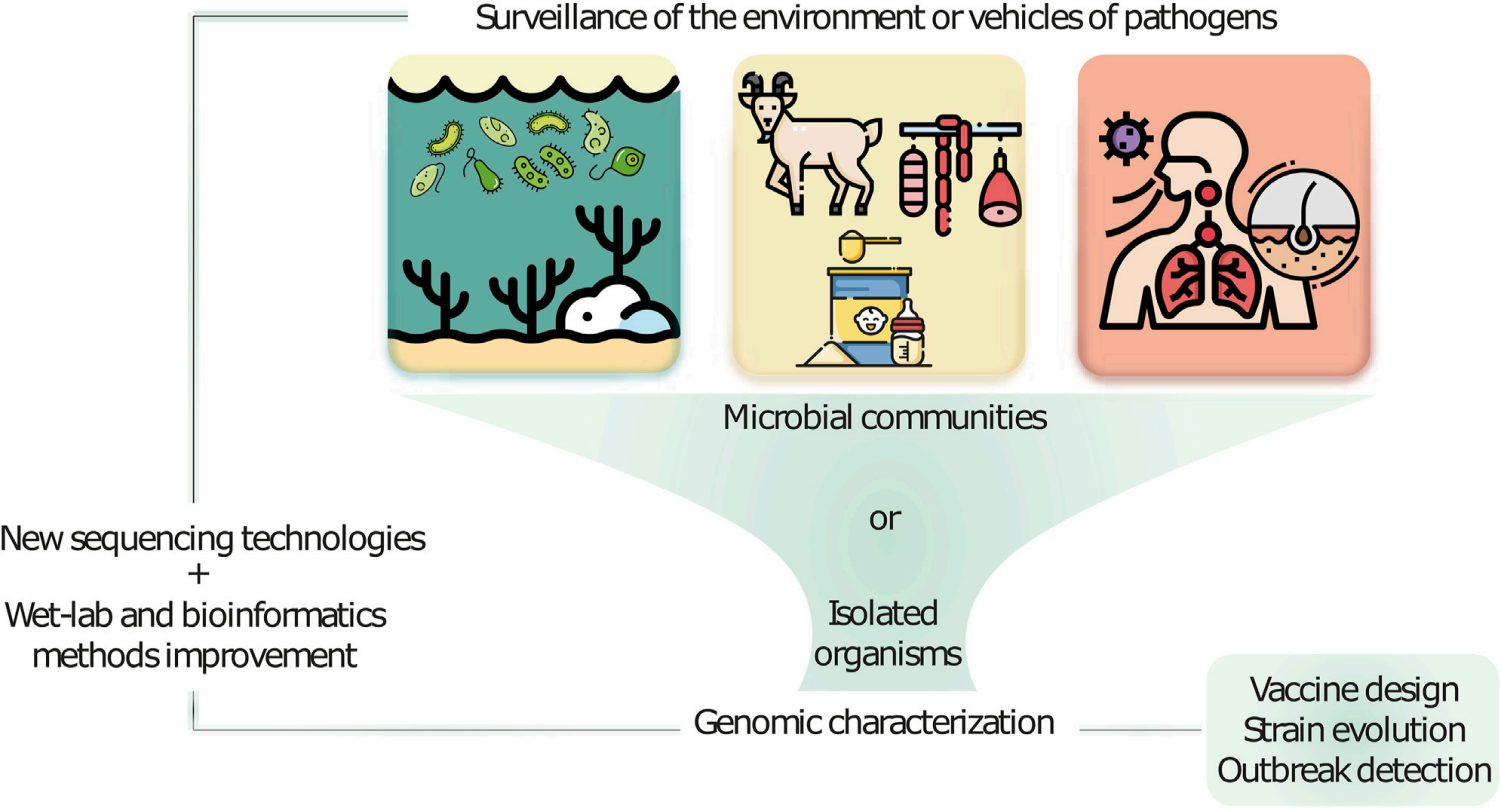
New sequencing technologies in combination with further omics approaches and improved wet-lab and bioinformatics development advance microbial characterization and molecular surveillance of samples taken from various environments.

This Research Topic collects contributions at the edge of the transition era to new sequencing technologies and their application to integrative omics in molecular surveillance (Figure 1). The selected articles address various areas of molecular surveillance in human health, animal health, food safety, and related fields using established short-read and novel long-read sequencing technologies as well as integration of multiple omics data sources. The included studies highlight the challenges, limitations, and future aspects of sequencing and multi-omics-based microbial characterization, particularly in the context of molecular surveillance.

Our journey into the era of new sequencing technologies begins with the monitoring of microbial communities in environmental samples. Studies focus on the detection of unknown microbes, the survey of the global distribution of known taxa, and the causes and implications of the composition of certain microbial communities. As an example, Ríos-Castro et al. use DNA metabarcoding to analyze environmental samples from sediment and marine water samples of a marine ecosystem. They identified pathogens with high relevance for the aquacultural sector and which are of medical and veterinary interest. This study demonstrates the potential of NGS technology for environmental monitoring. Although 16S high-throughput sequencing is widely used worldwide, most protocols do not take into account the variable biomass of microbes, which affects the relative bacterial abundances per sample. Here, Cruz et al. describe a novel equivolumetric protocol that generates library sizes proportional to the total microbial load in 16S rRNA amplicon sequencing. Therefore, the authors introduced 16S amplicon sequencing with short reads in combination with Bayesian cumulative probability models to address this issue and to ultimately achieve higher proportionality between library sizes and total microbial load.

Current NGS technology allows for in-depth characterization and molecular surveillance of certain bacterial pathogens. The authors of four studies in the Research Topic utilized different technologies to characterize outbreaks of these pathogens *via* bioinformatic approaches comprising pangenome comparisons, Multilocus Sequence Typing (MLST), Genomewide Association Studies (GWAS), and core-genome based phylogenies. All four studies involve at least short-read based Whole-Genome Sequencing (WGS) but also highlight the advantages and challenges of integrating data derived from technologies such as Oxford Nanopore long-read sequences (ONT). In the first study, Karthik et al. performed comparative genomics and WGS short-read sequencing of Indian isolates of *Brucella melitensis*, a zoonotic pathogen prevalent worldwide. *Via* pangenome analysis and MLST, the authors were able to predict several virulence genes and prophage regions and they were able to phylogenetically resolve different lineages of this species within India, where many isolates have been previously reported. The decreasing costs of NGS, open-source bioinformatics tools, and more easily operated laboratory technology make this approach more globally accessible for monitoring (Holzer et al., 2021; Khan et al., 2021). In another study, Parra-Flores et al. were able to give a detailed profile of virulence and antimicrobial resistance (AMR) proteins of *Cronobacter sakazakii* strains by adding MALDI-TOF-MS data to WGS data. The strains were isolated from powdered and dairy formulas and showed diverse virulence factors as well as resistance to beta-lactam antibiotics both *in silico* and *in vitro*. Their results reinforce a governmental decision to recall all involved powdered and dairy formulas in Chile in 2017 and further highlight the importance of continued molecular surveillance to detect product contamination by this pathogen. A third study deals with a foodborne pathogen, *Listeria monocytogenes*. Here, Chiaverini et al. performed phylogenetic analysis and GWAS on WGS short-read data to describe an outbreak due to this pathogen in Central Italy. Besides they were not able to identify any genomic differences between the outbreak and a reemergent strain, they found a possible differentiation by transcriptional factors and phage genes. Phages also play an important role in the dissemination of AMR genes of an unusual chimeric methicillin-resistant *Staphylococcus aureus* (MRSA) strain, as presented by Burgold-Voigt et al. In this study, the authors combined short-read WGS with long-read ONT sequencing to perform hybrid-assembly of an MRSA strain and its bacteriophages. By combining different sequencing technologies, they were able to shed light on several interesting features and the evolution of *S. aureus*.

Due to the novelty and rapid development of molecular technologies, there are still many challenges in data acquisition, standardization, and reproducibility; especially concerning downstream bioinformatics analysis (Hodcroft et al., 2021). This represents a challenge for wet-lab scientists, who are not trained in bioinformatics and need to process a huge amount of samples. Brandt et al. present an option to tackle this problem with an easy-to-use, fast, and robust workflow for the reconstruction of SARS-CoV-2 genomes, namely poreCov. The study highlights the importance of integrating bioinformatics software into reproducible pipelines using state-of-the-art workflow management systems and containers to finally bring new technologies into production and to allow the usage by non-experts (Hodcroft et al., 2021).

Within this Research Topic, we have assembled a diverse portfolio of studies on the molecular surveillance of bacteria, eukaryotic microbes, and viruses; analyzed using a variety of technologies and computational approaches. The results of these studies could impact important areas such as the development of new vaccines, government decisions on molecular surveillance of pathogens in food, control of pathogens in livestock, and methods for tracking outbreaks. In addition, we are particularly pleased to have attracted studies from researchers around the world: India, Chile, Italy, Spain, Germany, and Brazil; and we are grateful to all authors and reviewers for their contribution to this Research Topic.
